# Exploring the factors impacting physicians’ attitudes toward health information exchange with patients in Jordanian hospitals

**DOI:** 10.1186/s40545-023-00514-7

**Published:** 2023-01-17

**Authors:** Emad Dahiyat, Faris El-Dahiyat, Ghaleb El Refae, Zaheer-Ud-Din Babar

**Affiliations:** 1 College of Law, United Arab Emirates University, Al Ain, United Arab Emirates; 2 Clinical Pharmacy Program, College of Pharmacy, Al Ain University, Al Ain, United Arab Emirates; 3 AAU Health and Biomedical Research Center, Al Ain University, Abu Dhabi, United Arab Emirates; 4 College of Business, Al Ain University, Al Ain, United Arab Emirates; 5 Department of Pharmacy, School of Applied Sciences, University of Huddersfield, Huddersfield, HD1 3DH West Yorkshire UK

**Keywords:** Treatment decision-making, Hospitals, Physicians and patients, Communication

## Abstract

**Background:**

The uniqueness of the physician–patient relationship and the latter's lack of medical experience and knowledge necessitate providing patients with accurate and timely information necessary to engage them in treatment decision-making. Without detailed information from their physicians, patients cannot understand their medical condition, assess treatment options, and participate meaningfully in their care.

**Objectives:**

The present research determines the main factors influencing physicians' attitudes toward health information exchange with patients in Jordanian hospitals. The fundamental question addressed by this paper is why Jordanian physicians are reluctant to provide their patients with detailed health information about the potential risks, complications, and benefits of proposed treatments and other recognised alternative therapies.

**Method:**

This study is qualitative in nature, adopting face-to-face interviews as the key instrument of data collection in two hospitals in Jordan. The chief consideration of the sampling process was to select direct informants whose input would generate accurate results that might be generalised or translated to other contexts or settings. Thematic analysis was then used, and all participants’ opinions, answers, and interactions were transcribed and then reduced into themes and patterns for research, as per similarities and relationships, through coding and representing the data.

**Key findings:**

The findings show that most patients in government hospitals, especially those elderly, poorly educated, or inexperienced, choose practitioners based solely on medical service fees and costs rather than quality and convenience. On the other hand, the large number of patients attending public hospitals and the insufficiency of physicians' financial incentives in such hospitals may discourage physicians from providing patients with detailed health information. Matters, however, take a different turn in private hospitals, in which many physicians improve the patient experience to keep him and attract others by sharing information with patients about their health and treatment. However, it was noted that some physicians at such hospitals still rely heavily on their relations with health insurance companies to attract patients rather than on meaningful communication with their patients. Finally, the present findings reveal that the absence of a clear legal duty of medical disclosure negatively influences the amount of information received during the clinic visit.

**Conclusions:**

The fact that the level of communication in Jordanian healthcare settings has not been determined in detail opens the door to unnecessary healthcare expenditure and creates uncertainty concerning the amount of information that patients need to know in order to be involved in their treatment decision-making. The lack of proper control and quality monitoring may also negatively affect the interests of patients and their rights to receive adequate information about their health status.

## Introduction

Effective physician–patient communication is regarded as a pressing need by practitioners and policymakers throughout the world. In addition to enhancing patient involvement in treatment decision-making, information exchange between physicians and patients contributes to improving patient understanding of health conditions, increasing adherence to the treatment plan, and improving patient care outcomes [1]. However, enhancing physician–patient communication in Jordanian hospitals requires an effective health monitoring system, an adequate incentive package for physicians, and clear legal rules clarifying the extent and nature of the information that should be disclosed. Due to difficult economic conditions and mismanagement, the Jordanian government has become unable to effectively monitor the healthcare system or establish incentives for healthcare providers to increase quality and treat their patients appropriately. As Physicians enjoy an information advantage over patients concerning the appropriate treatment for the patient, it is highly possible that they use their informational advantage to increase personal income by ordering unnecessary tests or using irrelevant techniques or prescribing more expensive medicine despite the existence of cheaper generic medication [2, 3]. It is certain that an absence of information interaction between a patient and a physician can produce ineffective outcomes in healthcare systems and preclude a patient from receiving high-quality treatment at a reasonable cost.

The mere signing of a health care services agreement (informed consent) cannot always ensure and be used as evidence that patients have understood the information regarding treatment procedures and their possible risks. This is because patients usually do not read detailed medical information, but they only automatically sign the agreement to gain access to a medical service they seek, as they know that any refusal to sign will hinder any further meaningful access to a healthcare service. Even if patients read printed information in the agreement, they lack expert knowledge to understand it and assess the quality of care they receive. What complicates the matter further is that many elements of such agreement are implicit and, therefore, difficult to determine. Moreover, it is doubtful that this agreement represents a fair or balanced contract because it typically says nothing about its parties’ rights, interests, and obligations [4, 5]. Hence, it is closer to being a release of liability form or waiver of liability document rather than a two-sided contact. On the other hand, physicians do not usually function in a vacuum but rather as part of a complex team. Hence, the performance of individual physicians is difficult to measure because the individual’s output cannot be observed or distinguished from a team’s, especially during surgical operations. This is perhaps why it is difficult to prove medical mistakes and negligence, and this is also why many lawsuits against physicians fail. If patient protection is to be improved, then engaging patients in the medical decision-making process must be regulated more strictly by determining the requisite level of engagement so that any significant concealment or misrepresentation shall amount to gross medical malpractice, whether committed intentionally or negligently. Thus, we need smart regulation that ensures patients get a sufficient, accurate, and fair explanation concerning treatment plans, possible risks of medical treatment, and alternative medications. This implies that the disclosure of medical information shall not only be considered an ethical obligation, but also a legal obligation. This may also necessitate the documentation and reporting of the medical decisions and therapy steps and the public disclosure of information about healthcare service providers' quality and specific competence. This would not only serve transparency, but it will also assist patients in making decisions about their medical treatment while allowing service providers to reasonably raise prices to the level that covers the costs of improving quality. Without smart regulations and some form of external support, patients cannot choose their providers based on quality.

Several studies discussed the impact of communication between physicians and patients on the quality and cost of medical care [6–10]. Such studies show that stronger physician–patient relationships are correlated with improved patient outcomes. Although rapidly developing information technologies, like the Internet, can improve the patient's access to health information, many patients still lack the expertise or understanding to properly assess and interpret health information on the world wide web. It is worth noting at this point that the online environment is not a safe place nowadays, and it is difficult to always ensure the reliability of online information. What complicates the matter further is the fact that monitoring information flowing into and out of the network is extremely difficult, if not impossible, especially in light of the massive growth of social media, live chat, online forums, and other tools that could be exploited to spread misleading medical information about diseases. Hence, physicians are still the most reliable source of medical information.

This study aims to explore factors that influence asymmetric information in governmental and private hospitals in Jordan from the perspective of physicians, patients, legal consultants, and healthcare managers.

## Methods

### Research methodology

This study is qualitative in nature, adopting face-to-face interviews as a method to gather data from two governmental and private hospitals in Jordan. A convenience technique was employed to select patients, directors, and physicians for interviews. The selection of participants for the interviews was based on convenience and purposive sampling techniques, resulting in a total of 17 participants (see Table 1). The interviews listened to several times until a general idea was obtained. The validity of our qualitative research was checked by a technique known as respondent validation. This technique involves testing initial results with participants to see if they still ring true.

**Table 1:** Numbers of interviewees in the case study

Category	Number of interviewees
Patients	7
Directors	4
Physicians	6

### The study sample

The sample interviewed consisted of patients, physicians, legal directors, and medical directors. Two participants were females, and all the others were males, working in different departments of the selected hospitals. Participants’ opinions, answers, and interactions were transcribed, and then reduced into themes and patterns for analysis, as per similarities and relationships, through a process of coding and representing the data. The identities of the interviewees were not revealed for reasons of privacy and confidentiality. A brief profile of each interviewee is presented in Tables 2, 3, 4.

**Table 2:** Participant profiles: patients

Participant no.	Gender	Age (years)	Educational level	The monthly income
1	Female	70	No formal education	Less than 500 JD
2	Female	40	Diploma	Less than 500 JD
3	Male	55	Postgraduate	More than 1000 JD
4	Male	65	Bachelor degree	More than 500 JD
5	Male	39	Bachelor degree	More than 1000 JD
6	Male	68	Diploma	More than 2000 JD
7	Male	61	Primary school	Less than 500 JD

**Table 3:** Participant profiles: physicians

No.	Designation	Gender	Age	Qualifications	Years of experience
1	Ear, nose, and throat physician	Male	32	Bachelor	8
2	Nephrologist	Male	58	Bachelor	30
3	Gastroenterologist	Male	41	Bachelor	15
4	General practitioner	Male	35	Bachelor	10
5	Oncologist	Male	39	Bachelor	14
6	Internist	Male	38	Master	11

**Table 4:** Participant profiles: directors

No.	Designation	Gender	Age	Qualifications	Years of experience
1	Administrative manager	Male	48	Bachelor	20
2	Director of the legal affairs department	Male	42	Bachelor	15
3	Legal consultant	Male	33	Master	10
4	Administrative manager	Male	45	Bachelor	16

### Data gathering and analysis

Face-to-face interviews were conducted to collect data from Jordanian patients who were targeted by visiting the two hospitals in this investigation. The interview questions were centred on the main factors in Jordanian hospitals affecting communication between physicians and their patients. The duration of the interviews varied between 20 and 30 min. Likewise, the participants were informed that they had the right to withdraw from the study at any time. The audio-recorded interviews were transcribed verbatim, and the transcripts were reviewed by the authors. Thematic analysis was then used, and four themes were generated. During the analysis phase, the authors examine the data that have been collected in search of trends, themes and subthemes. After deriving primary themes and results, the authors assess the accuracy of such results in light of the participants’ feedback and answers.

## Results

The data analysis revealed the following key themes related to the factors influencing physicians' attitudes toward health information exchange with patients in Jordanian hospitals, as described by the respondents during the interviews.

### Socio-economic status of patients

The patient's socioeconomic status was the first factor to emerge as a theme in its effect on information asymmetries. Socio-economic status (SES) is a complex term that generally encompasses not only income and education level, but also a wide range of associated factors such as occupation, housing, and living environment. In the present study, it was found that of the patients above 60 years, 75% have not communicated with their physicians about their medical preferences or health conditions, compared to 25% below the age of 60 years. It was also noted that poorly educated patients (primary school or no formal education) are generally not interested in obtaining information about the quality of healthcare provider or physician qualifications or the details of illness and treatment plan. They even did not know the purpose of their medications and believe that their physicians know the best medical choices better than they do. This was exemplified in the following quotations from interviewees:

*“I trust that my physician is in the best position to weigh the risks and benefits associated with medical treatment” *(Interviewee P1).

*“I don’t want to get involved in complicated medical details that I don't understand”* (Interviewee P7).

The same opinion was mostly reported by low-income patients whose monthly income is below 1000 JD and those who pay for medicines and have many medications in their repeated prescriptions. According to such patients, the cost was an important factor when selecting their physicians regardless of the quality of healthcare services or the attitudes of their physicians. Their responses included the following statements:

*“Given that my health insurance does not cover all health costs, I just choose a lower price physician who can understand my financial and health circumstances”* (Interviewee P2).

*“My monthly salary is barely meeting my family's needs. This is why I only communicate with my physicians about the price of my medicine rather than the details of the treatment plan” *(Interviewee P4).

The matter, however, takes a different turn in the case of higher-educated respondents who emphasised the desirability of having involved in medical decision-making regarding their health. They believe that their involvement will improve their adherence to therapeutic plans. They also think that patients know their own medical preferences better than their physicians do. Likewise, the results show that high-income patients care more about achieving the best treatment regardless of the cost. They are generally interested in obtaining full information about the quality of healthcare providers and physician qualifications and reputation.

The above findings were derived from the following quotes extracted from the interviews with some patients.

*“Of course, I am interested in obtaining information tailored to my own health situation. For example, I need a full explanation of how my blood sugar or glucose result compares with the normal range, what the consequences are, and how I could take steps to favourably affect the results” *(Interviewee P3)*.*

*“Sharing all information with my doctor and receiving all details about my health conditions were particularly helpful in motivating me to adhere to prescribed therapy”* (Interviewee P5).

*“I want my physician to talk to me in depth and discuss my health issues to ensure I fully understand my medical condition and all available options. This necessitates visiting a well-known clinic which has highly qualified physicians”. *(Interviewee P6).

### Adequacy of human and financial resources

The second factor to emerge as a theme, in its effect on information interaction between a patient and a physician, was the adequacy of human and financial resources. The majority of the physicians interviewed at the public hospitals mentioned that the ministry of health has to recruit sufficient numbers of adequately trained physicians to accommodate patient needs and adapt to the increasing number of patients, especially patients with chronic diseases. The interviewees also agreed that having a sufficient number of suitably competent physicians is typically associated with high-quality patient outcomes. This was exemplified in the following quotes extracted from the interviews with physicians working at the sampled public hospital:

*“The staff shortage in this hospital surely affects our performance and the quality of care provided to patients. In my view, explaining all details for patients is next to impossible if you see more than 50 patients a day”. *(Interviewee 1).

*“In my own practice recently, I had a 10-h day and 60 patient contacts. I have great difficulty remembering my patients’ details or concerns. I am really overwhelmed by my workload, and this is why I don’t know how to overcome my Fear of Making Mistakes or missing important remarks, which could lead to medical errors and negligence”. *(Interviewee 3).

*“Working long hours with high numbers of patients is leaving no time for me to listen carefully to my patients, focus on the right symptom, or even discuss their treatment plan with them”. *(Interviewee 4).

While physicians practising in private hospitals usually have time to talk more with their patients, physicians at public hospitals deal with a high number of patients, and hence they may find less time to listen or speak with their patients. 20% of the interviewees working as physicians at the sampled public hospital strongly agreed with the statement: “I can provide sufficient time to all of my patients,” 65% disagreed or strongly disagreed, and 15% were neutral with the statement.

On the other hand, the data analysis found that most respondents emphasised competitive salaries, benefits, and incentives as the main factors impacting physicians’ practice and interactions with patients. Most interviewees highlight the need for hospitals to offer their physicians high salaries and rewards to motivate them effectively and positively to provide patients with sufficient care, information, and awareness. This need is becoming more urgent in public hospitals where physicians obtain the same low salary despite the currency depreciation and rising inflation in recent years. This does not necessarily mean that matters are always perfect in private hospitals. Insurance coverage may interfere with the functioning of healthcare markets, giving price considerations precedence over treatment quality. Often the patient chooses a physician from the insurance company’s list based only on price rather than quality or convenience. At the same time, insurance companies used to negotiate payment rates without regard to physician’s attitude towards patients. This may disappoint many individual physicians at private clinics and prevent markets from achieving competitive equilibria.

Most of the responses echoed the following interview extracts:

*“In my opinion, the support we get as physicians in this hospital will surely enhance the performance of physician staff and improve our interactions with patients. This will also lead to increased job satisfaction”. *(Interviewee 2)*.*

*“I think it is difficult for physicians to provide high levels of care quality if there is a shortage of funds. Because of high salaries, benefits and compensation at our private hospital, compared to other hospitals in Jordan, we do everything we can to provide patients with a positive experience, full information, and comprehensive assessment”. *(Interviewee 5)*.*

*“The sole reliance of some private physicians on their relationships with insurance companies to meet their financial needs may create little pressure to increase clinical quality in order to attract new patients. This will negatively affect the interests of patients and their rights to receive proper treatment and adequate information about their health status”. *(Interviewee 6).

### Administration’s control and external monitoring

Monitoring and controlling performance are considered one of the most important managerial functions. Monitoring is about setting standards, gathering data, evaluating against standards, and taking remedial actions that will enable hospitals to improve their team’s performance. Monitoring physicians’ performance is also necessary to assess the patient’s experience of care during their stay in the hospital by examining the communication patterns between physicians and patients and the extent to which decision-making is shared between both parties. This can be done through a survey distributed to the patients focusing on how well physicians communicate and how understandable their explanations are. Unfortunately, there is no mandatory reporting requirement or systematic monitoring in many Jordanian hospitals, and international accreditation is not explicitly required. One of the significant challenges is the absence of any national ranking for the hospitals or physicians in Jordan based on their attitudes toward patients or the quality of their care.

The data analysis revealed that most of the directors interviewed cited sufficient monitoring and control as the key factor that reduces physician misbehaviour and helps healthcare markets function more effectively. The administrative managers interviewed declared that a large percentage of physicians in private hospitals are not closely subject to direct and continuous supervision from the hospital, as they are merely tenants of clinics in the hospital and have no employment contract with the hospital. Also, they mentioned that the culture of external quality assurance is not well established in Jordanian public hospitals, despite the importance of external quality assurance in enhancing the quality of care and the active disclosure of information by evaluating service providers and their quality of care and by appropriate reporting of the results and documentation of medical activities.

The following statements reflect the interviewees’ views concerning this finding (theme):

*“Inappropriate monitoring had a negative effect on both organisational effectiveness and individual effectiveness. Introducing effective monitoring of clinicians’ practice may improve clinical productivity and doctors’ attitudes towards their patients”. *(Interviewee D2)*.*

*“For my part, when I see that my managers monitor my performance and appreciate my patients’ notes and other documented results, I do my utmost to give my best”. *(Interviewee 6)*.*

*“The role of the hospital has changed into an economic institution that only cares about its own investment. This makes it unable to closely monitor the doctor’s effort level, and hence this may lead to a higher risk of professional misconduct. In many cases, physicians are merely seen as tenants of clinics at the hospital”.* (Interviewee D1).

*“The medical performance in my hospital is either poorly or not managed by the hospital’s administration. There is no formal evaluation process for the physician’s performance and attitude unless they have an administrative position at the hospital or the ministry.” *(Interviewee 1).

*“I see very little feedback or evaluation of the performance of doctors in my department. We never heard about accreditation, credentialing or the self-assessment process”. *(Interviewee 4).

### The adequacy of the legal framework governing the physician–patient relationship

The legislation mainly aims to impose minimum licensure qualifications to practise medicine and prohibit the unauthorised practice of medicine. However, there is no legal guarantee that licensed physicians will not use their informational advantage for personal gain. Nothing in the law obliges the physician to record his decisions in writing to be verified later, and there is no legal provision that specifies the amount of information the doctor must disclose to his patient. According to Jordanian Law, the physician–patient relationship shall be governed by an agreement clarifying the medical treatment’s scope. It should be noted that most healthcare agreements are not written down. This does not affect the validity of the agreement as the informed consent of patients can be in the form of implied consent, especially in low-risk therapeutic cases. Nevertheless, written consent should be used in high-risk cases, such as surgery, where oral consent is impossible. It should be noted here that Articles 7 and 8 of the Jordanian medical liability Law No.25 of 2018 provide that physician is prohibited from treating the patient without his consent, with the exception of cases that require emergency medical intervention and it is not possible to obtain approval. This implies that the physician has to inform a patient of the nature of his illness unless the patient’s psychological state or health condition does not allow him to be informed personally [11].

The physician also needs to disclose enough about the risks and benefits of proposed treatments so that the patient becomes sufficiently informed about and involved in their care. However, because Jordanian legislation does not specify the amount of information that must be disclosed, physicians might not be aware of what they must typically disclose. Traditionally, courts around the world held that a physician’s duty to disclose information to the patient depends upon community disclosure standards and what a reasonable person in the patient’s position would find important [12]. Physicians are thus required to disclose all information that might affect a patient’s treatment decisions, including the nature and character of the proposed treatment or surgical procedure, anticipated risks or benefits involved in the treatment or surgical procedure, and the alternative forms of treatment, including non-treatment.

According to the legal consultants interviewed at the sampled hospitals, there are no known rules about the scope of information to be disclosed by physicians. Under the existing legal framework, physicians are only obligated to disclose the relevant information whenever requested to do so. The current law does not directly mention the self-disclosure duty. Hence, courts in Jordan are likely to reject a claim for fraud based on the omission or non-disclosure of superior knowledge, especially if there is no evidence that physicians have an affirmative duty under the contract or the provisions of law to disclose.

The following statements reflect the interviewees’ views about this finding (theme):

*“In my opinion, healthcare agreement cannot ensure that patients have received clear information about their health conditions and cannot guarantee that patients will not be misled or exploited. The physician-patient relationship is not merely a contractual relationship. It also includes some kinds of reciprocal relationships based on trust and confidence. It is then ethically essential for the physician to disclose substantial information that may be reasonably relevant to a patient in making an informed decision relating to his medical choices”.*

*“Healthcare providers are not the guarantors of patients’ full recovery. As a result, not all medical failures can be sued. The lack of a clear legal or professional definition of mandatory and voluntary disclosure in the healthcare sector may lead to unethical medical practices”.*

## Discussion

The present study has shown that patients’ socioeconomic status (SES) influences health care quality and the amount of information received during the clinic visit. Patients with low SES do not communicate with their physicians about their medical preferences and possible treatment scenarios. This result corroborates those of other studies [4, 13] showing how socioeconomic factors affect patient experiences on quality of care and the amount of information presented by the physicians. Patients requiring more details come from higher social, economic, and educational statuses. The present findings reveal that staff and resource adequacy as well as good salaries and incentives represent the chief factor that improves the quality of health care and leads to better outcomes for patients. This result is similar to that of other studies, where it was confirmed that staff shortages might lead to excessive workloads, which in turn worsen the quality of healthcare, while adequate numbers of physicians help reduce individual workload and lead to patients receiving high-quality care and more time with their physicians [14, 15]. These findings also parallel other studies that have found that sufficient financial incentives can reduce the risk of offensive attitudes, motivate medical staff, and enhance their performance [16–19].

Additionally, according to the participants’ responses, the administration’s control and monitoring were recognised as essential factors in the current context. The present study has shown that monitoring may play a vital role in enhancing communication between physicians and their patients and improving healthcare quality. This result corroborates those of other studies [3, 20], indicating that monitoring is an effective method for reducing mistreatment in the healthcare context.

Finally, the present findings reveal that the absence of any specific duty on healthcare professionals to report or disclose all the details to the patients may create concerns about patients being exposed to deceptive treatment or misleading attitudes from some physicians. To protect patients against the absence of transparency in medical interventions, the amount of physician’s disclosure must be obviously determined without leaving the details to be governed by individuals’ arrangements or medical norms. This result is similar to that of other legal studies [21, 22], which explicitly require disclosure where one party possesses superior knowledge of material facts unknown to the other party or when the parties stand in a fiduciary relationship with each other. In some jurisdictions, non-disclosure may provide grounds for concealment fraud whenever a relationship of trust exists between the two parties [23].

## Conclusion

The Jordanian medical sector is suffering from many drawbacks including the inadequacy of human and financial resources, the weakness of the health monitoring system, and the absence of a legal framework governing medical disclosure. In fact, it is still unclear the extent to which physicians are obliged to disclose all relevant medical information to patients, and it is still uncertain what will constitute mandatory disclosure in Jordanian healthcare settings.

The present study has adopted face-to-face (F2F) interviewing whereas a quantitative approach could also confirm and support the results of the study and build on them through further work on how to reduce information asymmetry in Jordanian hospitals. One limitation of this study is the small sample as it only examined two governmental and private hospitals in Jordan without shedding light on the reality of the military hospitals of the Royal Medical Services or on other NGO hospitals in Jordan. As a result, the authors highly recommend a further examination of the issue, recruiting participants from other military sectors and NGO hospitals. Besides, respondents from different fields and professional levels, such as policymakers, Jordanian Doctors Syndicate, and other healthcare providers, need to be sampled using different methodological techniques that are intended at exploring the factors impacting the knowledge imbalance between patients and physicians in Jordanian hospitals.

## Data Availability

The data that support the findings of this study are available from the corresponding author upon reasonable request.
